# Seven-year longitudinal change in risk factors for non-communicable diseases in rural Kerala, India: The WHO STEPS approach

**DOI:** 10.1371/journal.pone.0178949

**Published:** 2017-06-09

**Authors:** Thirunavukkarasu Sathish, Srinivasan Kannan, Sankara P. Sarma, Oliver Razum, Odile Sauzet, Kavumpurathu Raman Thankappan

**Affiliations:** 1 Achutha Menon Centre for Health Science Studies, Sree Chitra Tirunal Institute for Medical Sciences and Technology, Thiruvananthapuram, Kerala, India; 2 Centre for Population Health Sciences, Lee Kong Chian School of Medicine, Nanyang Technological University, Singapore; 3 Department of Epidemiology and International Public Health, School of Public Health, Bielefeld University, Bielefeld, Germany; Florida International University Herbert Wertheim College of Medicine, UNITED STATES

## Abstract

Nearly three-quarters of global deaths from non-communicable diseases (NCDs) occur in low- and middle-income countries such as India. However, there are few data available on longitudinal change in risk factors for NCDs in India. We conducted a cohort study among 495 individuals (aged 15 to 64 years at baseline) in rural Kerala state, India, from 2003 to 2010. For the present analysis, data from 410 adults (aged 20 to 64 years at baseline) who participated at both baseline and follow-up studies were analyzed. We used the World Health Organization STEPwise approach to risk factor surveillance for data collection. Age-adjusted change in risk factors for NCDs was assessed using the mixed-effects linear regression for continuous variables and the generalized estimating equation for categorical variables. The mean age of participants at baseline was 41.6 years, and 53.9% were women. The mean follow-up period was 7.1 years. There were significant increases in weight (mean change +5.0 kg, 95% confidence interval [CI] 4.2 to 5.8), body mass index (mean change +1.8 kg/m^2^, 95% CI 1.5 to 2.1), waist circumference (mean change +3.9 cm, 95% CI 3.0 to 4.8), waist-to-height ratio (mean change +0.022, 95% CI 0.016 to 0.027), current smokeless tobacco use (men: odds ratio [OR] 1.6, 95% CI 1.1 to 2.2), alcohol use (men: OR 2.6, 95% CI 1.9 to 3.5; women: OR 4.8, 95% CI 1.8 to 12.6), physical inactivity (OR 2.0, 95% CI 1.3 to 3.0), obesity (OR 2.2, 95% CI 1.7 to 2.8), and central obesity (OR 1.9, 95% CI 1.5 to 2.3). Over a seven-year period, several NCD risk factors have increased in the study cohort. This calls for implementation of lifestyle intervention programs in rural Kerala.

## Introduction

Every year an estimated 38 million deaths occur globally due to non-communicable diseases (NCDs), namely cancer, cardiovascular disease, chronic respiratory diseases, and diabetes. Nearly three-quarters of these deaths occur in low- and middle-income countries (LMICs) such as India [1]. Routine surveillance of lifestyle risk factors for NCDs such as tobacco use, harmful use of alcohol, low fruit and vegetable intake, physical inactivity, obesity, and hypertension in a population, will help in monitoring trends and in turn will help in designing and monitoring interventions, policy planning, and allocation of health care resources [1,2]. The World Health Organization (WHO) developed the STEPwise approach to risk factor surveillance (STEPS) to collect, analyze and monitor within-country and between-country changes in risk factors for NCDs [2]. The WHO STEPS approach ensures data comparability over time using standardized questions, protocols and tools [2].

In India, repeat cross-sectional surveys have shown an increase in the prevalence of NCDs and its risk factors [3–5]. However, these repeat surveys have inherent limitations: (i) data from different regions of the country or within a state were compared whereas it would be more accurate to compare change within the same population over time; (ii) sampling strategies for repeat surveys were slightly different, reducing the validity of comparisons; (iii) the focus was mostly on disease (e.g. diabetes), rather than on risk factors. Measuring risk factors is easier and less resource intensive than diagnosing disease. This is particularly important for resource constrained settings such as India; and (iv) only a few studies were conducted in rural areas. Compared to repeat cross-sectional surveys, cohort studies provide valid and reliable estimates of risk factor changes in a population.

Epidemiological and demographic transitions in the Indian state of Kerala are more advanced than elsewhere in the country [6]. Kerala is therefore said to be the “harbinger” of the future burden of NCDs in India [7,8]. Kerala has also undergone a rapid nutritional transition in recent years [9] with studies showing that it has one of the highest rates of some major NCD risk factors in India [7,8]. In this study, we assessed the longitudinal change in risk factors for NCDs in rural Kerala using the WHO STEPS approach.

## Materials and methods

### Study design and participants

**Fig 1:**
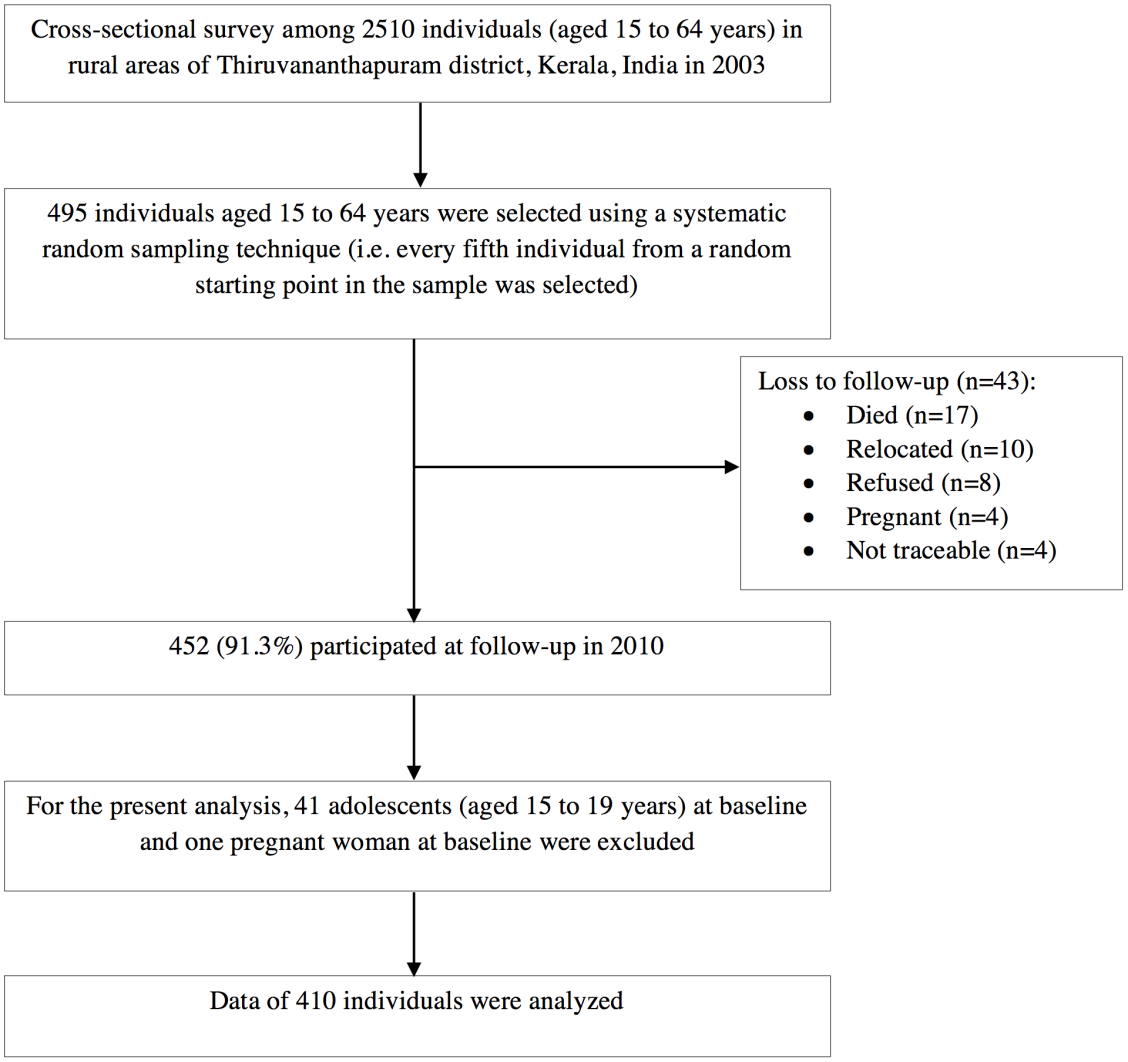
Flowchart showing the formation of the study cohort.

The study design has been described in detail elsewhere [7,8,10–13]. Briefly, in 2003, a cross-sectional survey was conducted among 2510 individuals (aged 15 to 64 years) using the WHO STEPS approach in rural areas of Thiruvananthapuram district, Kerala State, India [7]. One of Thiruvananthapuram district’s 19 *blocks* (administrative unit below district) was selected randomly and one of this block’s six *panchayats* (administrative unit below block) was chosen randomly. From this *panchayat*, 8 wards from a total of 15 wards were randomly selected, and households in the selected wards were approached to obtain a sample of 2510 individuals (aged 15 to 64 years). From this cross-sectional survey sample, a systematic random sampling technique (i.e. every fifth individual from a random starting point in the sample was selected) was used to select a sub-sample of 495 individuals (aged 15 to 64 years). Four hundred and fifty-two (91.3%) of these participated in the 2010 follow-up study. The reasons for the loss to follow-up of 43 study participants were: died (n = 17), relocated (n = 10), refused (n = 8), pregnant (n = 4), and not traceable (n = 4). There were no significant differences in any of the baseline characteristics between those followed-up (n = 452) and those lost to follow-up (n = 43) (data not shown). Among those who were followed-up, adolescents aged 15 to 19 years at baseline (n = 41) and one woman who was pregnant at baseline were excluded, leaving 410 individuals for the present analysis. The flowchart showing the formation of the study cohort is given in Fig 1.

### Ethics approval

The ethics committee of the Sree Chitra Tirunal Institute for Medical Sciences and Technology, Thiruvananthapuram district, Kerala, gave ethical clearance for both the baseline and follow-up studies (Approval No. Baseline study: SCTIEC-5/2003 and Follow-up study: IEC/ 271/May 08, 2010).

### Data collection

For both baseline and follow-up studies, trained staff administered a pre-tested and modified WHO STEPS questionnaire to collect data on demographic details, smoking, smokeless tobacco use, alcohol use, intake of fruits and vegetables, physical activity and use of medications. Physical measurements were taken in accordance with the WHO recommendations using standardized tools [2]. Body mass index (BMI) was calculated as weight in kilograms divided by the square of the height in meters (kg/m^2^). The waist-to-height ratio was calculated as the ratio between the waist circumference and the height. Blood pressure (BP) was measured using an *Omron* digital automatic BP monitor from the right arm in a seated position after having rested for at least five minutes. Two BP readings were obtained initially and a third reading was taken only if there was a difference of more than 10 mmHg in systolic or diastolic BP between the initial two readings. Accordingly, the average of two or more readings was considered as the participant’s BP.

### Definitions

Current smoking was defined as smoking any tobacco products such as cigarettes, *bidis* (hand-rolled cigarettes), cigars, or hookahs (water pipes) in the last 30 days [2]. Current smokeless tobacco use was defined as use of smokeless tobacco products such as *gutka* (a mixture of tobacco, lime and areca nut), *khaini* (a mixture of tobacco and lime), snuff and betel quid with tobacco in the last 30 days [2]. Alcohol users were those who drank at least one standard drink of alcohol (30 ml of spirits, 285 ml of beer or 120 ml of wine) in the last 12 months [2]. Physical inactivity was defined as no self-reported moderate or vigorous physical activity at work and leisure, and not walking or bicycling for travel. Overweight was defined as BMI ≥ 23 kg/m^2^ but <25 kg/m^2^, obesity as BMI ≥ 25 kg/m^2^, and central obesity as waist circumference ≥ 90 cm for men and ≥ 80 cm for women according to the WHO Asia Pacific guidelines [14]. Based on the Joint National Committee 7 criteria [15], hypertension was defined as systolic BP ≥ 140 mmHg and/or diastolic BP ≥ 90 mmHg, and/or currently using anti-hypertensive medications. Hypertension treatment was defined as use of anti-hypertensive medications [15]. Control of hypertension was defined as systolic BP<140 mmHg and diastolic BP<90 mmHg, and use of anti-hypertensive medications [15].

### Statistical analyses

Continuous variables are summarized using mean ± standard deviation, and categorical variables using frequency and percentage. The baseline characteristics of participants and non-participants at follow-up were compared using *t* test for continuous variables, and *χ*^2^ test or the Fisher’s Exact test (for 2×2 contingency table with cells less than five) for categorical variables. The mean change in continuous variables from baseline to follow-up was assessed using the mixed-effects linear regression with maximum likelihood parameter estimation by including age as a covariate in the model. For BP, in addition to age, hypertension treatment and control rates were included in the model. The change in categorical variables was examined by including age as a covariate in a binomial logistic regression model, with odds ratios (ORs), 95% confidence intervals (CIs) and p values estimated using the generalized estimating equation (logit link function) with an exchangeable working correlation matrix and robust standard errors. Clustering of risk factors (<2, 2–3 and 4–8 risk factors) was calculated using current smoking, current smokeless tobacco use, alcohol use, no daily intake of fruits and vegetables, physical inactivity, overweight or obesity, central obesity, and hypertension. The differences in clustering of risk factors between baseline and follow-up were tested using the logistic regression, adjusting for age. All p values were based on two-tailed tests of significance. P value less than 0.05 was considered statistically significant. Data were analyzed with STATA software (version 14.2; Stata Corp LP, College Station, TX, USA).

## Results

The follow-up study was conducted after a mean of 7.1 years (range: 6.7 to 7.5 years). Table 1 shows the baseline demographic characteristics of the study cohort. The mean age of participants was 46.1 years, more than half (53.9%) were women, and slightly more than 50% had 10 or more years of schooling. Most men (79.9%) were involved in unskilled or skilled labour, and the majority of women (80.1%) were housewives.

**Table 1:** Baseline demographic characteristics of the study cohort.

		N = 410
Age (years), mean ± SD		41.6 ± 12.8
Women, n (%)		221 (53.9)
Years of schooling, n (%)		
	<10	203 (49.5)
	≥10	207 (50.5)
Occupation, n (%)		
	Unskilled/Skilled labour	176 (42.9)
	Housewife	177 (43.2)
	Student	14 (3.4)
	Retired	12 (2.9)
	Unemployed	23 (5.6)
	Others	8 (2.0)

SD, standard deviation.

### Change in risk factors for NCDs

**Fig 2:**
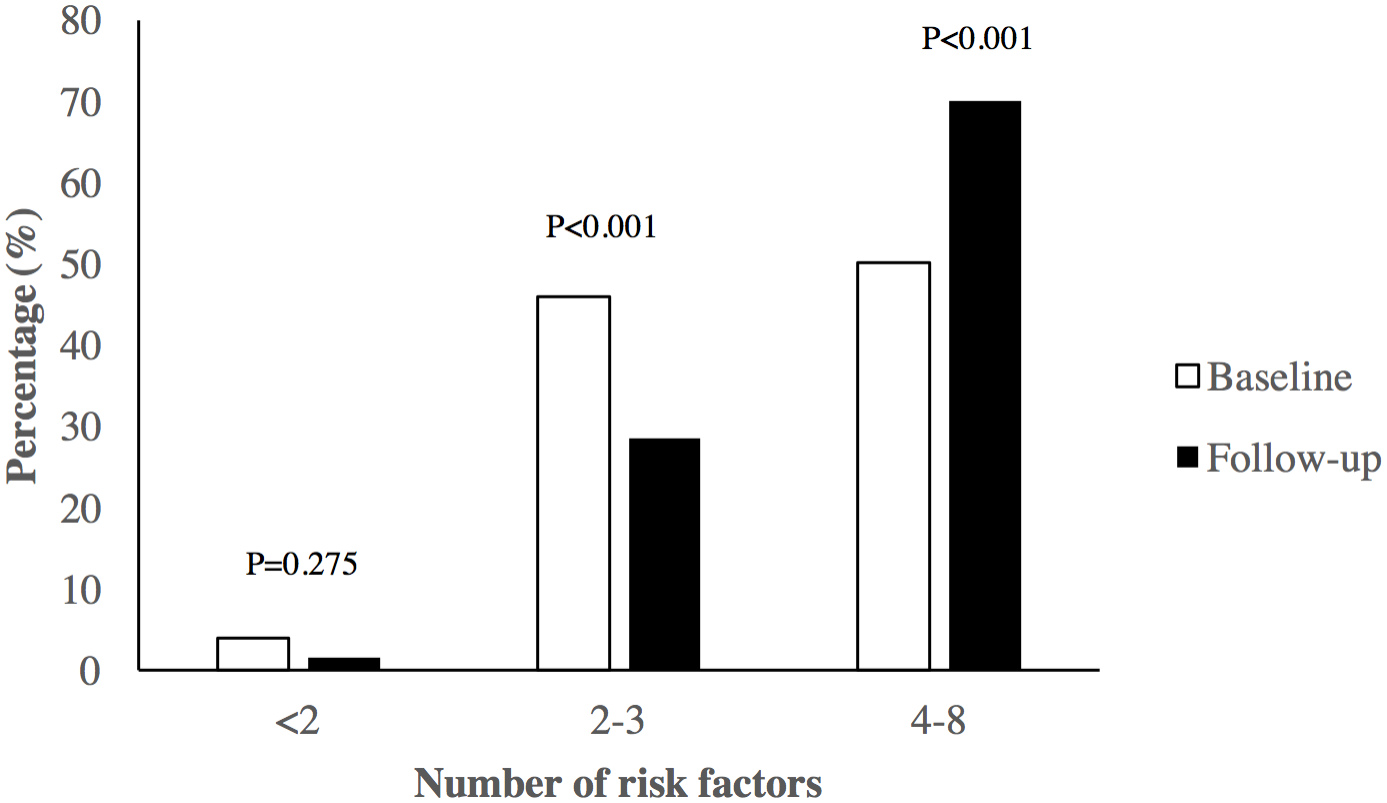
Age-adjusted clustering of risk factors for non-communicable diseases in baseline and follow-up studies.

Tables 2 and 3 show the age-adjusted change in NCD risk factors in the study cohort. BMI rose significantly by 1.8 kg/m^2^, resulting in a 120% (OR: 2.2) increase in obesity prevalence. Waist circumference rose significantly by 3.9 cm, resulting in a 90% (OR: 1.9) increase in central obesity prevalence. The prevalence of physical inactivity rose by 100% (OR: 2.0). Among men, there was a significant increase in the prevalence of current smokeless tobacco use by 60% (OR: 1.6) and alcohol use by 160% (OR: 2.6). Among women, the prevalence of alcohol use increased nearly five times from baseline to follow-up. The mean number of risk factors rose from 3.5 to 4.2 (p<0.001), and there was a significant increase in the proportion of individuals with four or more risk factors (Fig 2).

**Table 2:** Age-adjusted mean change in risk factors for non-communicable diseases in the study cohort.

	N	Baseline (2003)	N	Follow-up (2010)	Age-adjusted mean change^a^ (95% CI)	p value
Weight (kg)	410	55.0 ± 10.5	410	59.7 ± 11.4	5.0 (4.2 to 5.8)	<0.001
Body mass index (kg/m^2^)	410	22.4 ± 3.9	410	24.3 ± 4.3	1.8 (1.5 to 2.1)	<0.001
Waist circumference (cm)	410	84.5 ± 11.0	410	89.5 ± 11.1	3.9 (3.0 to 4.8)	<0.001
Waist-to-height ratio	410	0.541 ± 0.080	410	0.573 ± 0.078	0.022 (0.016 to 0.027)	<0.001
Systolic BP (mmHg)^b^	410	131.5 ± 18.6	410	134.8 ± 21.0	-0.3 (-2.2 to 1.7)	0.797
Diastolic BP (mmHg)^b^	410	81.1 ± 11.2	410	82.1 ± 10.1	0.1 (-1.0 to 1.3)	0.831

CI, confidence interval; BP, blood pressure.

Data are mean ± standard deviation or mean change (95% CI).

^a^ Estimated using the mixed-effects linear regression by including age as a covariate in the model.

^b^ Adjusted for treatment and control rates of hypertension, in addition to age.

**Table 3:** Age-adjusted change in the prevalence of risk factors for non-communicable diseases in the study cohort.

		N	Baseline (2003)	N	Follow-up (2010)	Age-adjusted odds ratio^a^ (95% CI)	p value
Current smoking^b^	Men Women	189221	65 (34.4)0	189221	63 (33.3)0	0.8 (0.6 to 1.1)--	0.148 --
Current smokeless tobacco use^c^	Men Women	189221	35 (18.5)12 (5.4)	189221	52 (27.5)20 (9.1)	1.6 (1.1 to 2.2)1.2 (0.7 to 2.3)	0.012 0.546
Alcohol use^d^	Men Women	189221	88 (46.6)5 (2.3)	189221	128 (67.7)23 (10.4)	2.6 (1.9 to 3.5)4.8 (1.8 to 12.6)	<0.001 0.001
No daily intake of fruits and vegetables		410	369 (90.0)	410	355 (86.6)	0.7 (0.5 to 1.1)	0.127
Physical inactivity^e^		410	337 (82.2)	410	372 (90.7)	2.0 (1.3 to 3.0)	0.002
Overweight^f^		410	73 (17.8)	410	67 (16.3)	0.9 (0.6 to 1.2)	0.440
Obesity^g^		410	97 (23.7)	410	172 (42.0)	2.2 (1.7 to 2.8)	<0.001
Central obesity^h^		410	204 (49.8)	410	276 (67.3)	1.9 (1.5 to 2.3)	<0.001
Hypertension^i^		410	150 (36.6)	410	177 (43.2)	0.9 (0.7 to 1.2)	0.572

CI; confidence interval.

Data are n (%) or odds ratio (95% CI).

^a^ Estimated using the generalized estimating equation by including age as a covariate in a binomial logistic regression model with an exchangeable working correlation matrix and robust standard errors.

^b^ Smoked any tobacco products in the last 30 days [2].

^c^ Used any smokeless tobacco products in the last 30 days [2].

^d^ Drank at least one standard drink of alcohol (30 ml of spirits, 285 ml of beer or 120 ml of wine) in the last 12 months [2].

^e^ No history of moderate or vigorous physical activity at work and leisure, and do not walk or bicycle for travel [2].

^f^ Body mass index ≥23 kg/m^2^ but <25 kg/m^2^ according to the World Health Organization (WHO) Asia Pacific guidelines [14].

^g^ Body mass index ≥25 kg/m^2^ according to the WHO Asia Pacific guidelines [14].

^h^ Waist circumference ≥90 cm for men and ≥80 cm for women according to the WHO Asia Pacific guidelines [14].

^i^ Systolic blood pressure ≥140 mmHg and/or diastolic blood pressure ≥90 mmHg and/or current use of anti-hypertensive medications according to the Joint National committee 7 criteria [15].

## Discussion

This is one of the first studies from a LMIC to use the WHO STEPS approach to assess the longitudinal change in risk factors for NCDs. Over a seven-year period, there were significant increases in weight, BMI, waist circumference, waist-to-height ratio, current smokeless tobacco use (in men), alcohol use (both sexes), physical inactivity, obesity and central obesity in the study cohort, after adjusting for age. This probably explains the high prevalence of diabetes [7] and high mortality due to cardiovascular disease in Kerala [16]. The increase in NCD risk factors in Kerala is paradoxical to its high literacy rate, high life expectancy, better indicators of socio-economic status, and better access to healthcare services [17]. Our study findings reflect the increase in unhealthy lifestyle behaviours in rural Kerala including consumption of energy-dense foods and low physical activity due to rapid urbanization and nutrition transition [9,18]. Disproportionate to the decreasing levels of physical activity, the per capita daily calorie intake in Kerala has increased by two-thirds over the last three decades [19]. People in Kerala consume twice the amount of processed food items high in salt and sugar compared to those in the rest of India [19]. In addition, foods in Kerala are rich in saturated fats and a very low percentage of Kerala’s population consumes adequate amounts of fruits and vegetables [20].

The increase in the prevalence of current smokeless tobacco use among men in our study is of public health relevance given that more than 50% of oral cancers in India are attributed to this exposure [21]. The increase in alcohol use in our study needs to be seen in the light that South Asians are at a higher risk for coronary heart disease with moderate to high alcohol intake [22], and one-fifth of drinkers in India fall into the criteria of dependent drinking and more than half fall into that of hazardous drinking [22]. The increase in alcohol use among women in our study is in line with observations in other parts of India [22] and may be related to advertising; however, further exploration is warranted.

Surprisingly, there was no significant change in mean BP or the prevalence of hypertension, despite a high incidence of hypertension in the study population [8] and after adjusting for hypertension treatment and control rates. Possible explanations for these findings include regression to the mean among participants with high BP [23] or an insufficient length of the follow-up period to elicit change [24].

Our study has a number of strengths. Firstly, we employed a prospective cohort design to assess the change in risk factors for NCDs, providing valid and reliable estimates compared with repeat cross-sectional surveys. Secondly, we used the WHO STEPS approach and standardized tools to measure NCD risk factors at both baseline and follow-up. The WHO STEPS approach enables within-country and between-country comparisons of change in risk factors over time [2]. Thirdly, our study had a high follow-up rate (91.3%) with no significant differences in baseline characteristics between participants and non-participants at follow-up, making selection bias unlikely. Finally, the data completeness was 100% at both baseline and follow-up.

Inevitably, however, our study has certain limitations. Firstly, we could not follow the cohort more than once due to limited resources. While the time period between the two contacts with participants was sufficiently long to assess change in most of the risk factors [2], we were not able to identify precisely when a change in behaviour occurred (e.g. onset of smoking). Secondly, our study sample size was relatively small, which might have resulted in a type II error, thereby failing to detect change in some risk factors. However, this is one of the first studies of its kind from a LMIC and we believe this will lead to future longitudinal studies with larger samples to validate our study findings. Thirdly, due to limited resources, we did not collect blood samples to estimate plasma glucose, HbA1c and serum lipids at follow-up. Furthermore, we did not collect data on use of medications for dyslipidemia and therefore change in those variables could not be assessed. Nevertheless, it can be reasonably assumed that the biochemical parameters could also have worsened, given the positive association (although modest) between anthropometry, and glycemic indices and lipid profile [7]. Of note, the WHO does not recommend blood sample collection to measure biochemical risk factors in resource-constrained settings [2]. Finally, our study was conducted in one rural area in Southern India. Therefore, the study findings may not necessarily be extrapolated to other parts of India or other LMICs. However, the state of Kerala is in the late stage of epidemiologic transition [6] and therefore change in NCD risk factors in this state is likely to reflect future change in most parts of India [7,8].

The burden of NCDs is rapidly increasing in every region of the world, particularly in LMICs. There is, therefore, a need to continuously monitor trends in NCD risk factors in countries such as India in order to be able to effectively control the global NCD epidemic. The magnitude of change for some risk factors in our study is alarming, given that an increase in BMI of 1.0 kg/m^2^ is associated with a 25% diabetes risk [25] and a 6% cardiovascular risk [26]. Therefore, our study findings call for urgent development and widespread implementation of lifestyle intervention programs in rural Kerala [27–29].
