# Correction: Seven-year longitudinal change in risk factors for non-communicable diseases in rural Kerala, India: The WHO STEPS approach

**DOI:** 10.1371/journal.pone.0183379

**Published:** 2017-08-10

**Authors:** The PLOS ONE staff

Table 3 appears incorrectly. Please see the complete, correct Table 3 here. The publisher apologizes for the error.

**Table 3:** Age-adjusted change in the prevalence of risk factors for non-communicable diseases in the study cohort.

		N	Baseline (2003)	N	Follow-up (2010)	Age-adjusted odds ratio^a^ (95% CI)	p value
Current smoking^b^	Men Women	189221	65 (34.4)0	189221	63 (33.3)0	0.8 (0.6 to 1.1)—	0.148 —
Current smokeless tobacco use^c^	Men Women	189221	35 (18.5)12 (5.4)	189221	52 (27.5)20 (9.1)	1.6 (1.1 to 2.2)1.2 (0.7 to 2.3)	0.012 0.546
Alcohol use^d^	Men Women	189221	88 (46.6)5 (2.3)	189221	128 (67.7)23 (10.4)	2.6 (1.9 to 3.5)4.8 (1.8 to 12.6)	<0.001 0.001
No daily intake of fruits and vegetables		410	369 (90.0)	410	355 (86.6)	0.7 (0.5 to 1.1)	0.127
Physical inactivity^e^		410	337 (82.2)	410	372 (90.7)	2.0 (1.3 to 3.0)	0.002
Overweight^f^		410	73 (17.8)	410	67 (16.3)	0.9 (0.6 to 1.2)	0.440
Obesity^g^		410	97 (23.7)	410	172 (42.0)	2.2 (1.7 to 2.8)	<0.001
Central obesity^h^		410	204 (49.8)	410	276 (67.3)	1.9 (1.5 to 2.3)	<0.001
Hypertension^i^		410	150 (36.6)	410	177 (43.2)	0.9 (0.7 to 1.2)	0.572

CI; confidence interval.

Data are n (%) or odds ratio (95% CI).

^a^ Estimated using the generalized estimating equation by including age as a covariate in a binomial logistic regression model with an exchangeable working correlation matrix and robust standard errors.

^b^ Smoked any tobacco products in the last 30 days [2].

^c^ Used any smokeless tobacco products in the last 30 days [2].

^d^ Drank at least one standard drink of alcohol (30 ml of spirits, 285 ml of beer or 120 ml of wine) in the last 12 months [2].

^e^ No history of moderate or vigorous physical activity at work and leisure, and do not walk or bicycle for travel [2].

^f^ Body mass index ≥23 kg/m^2^ but <25 kg/m^2^ according to the World Health Organization (WHO) Asia Pacific guidelines [14].

^g^ Body mass index ≥25 kg/m^2^ according to the WHO Asia Pacific guidelines [14].

^h^ Waist circumference ≥90 cm for men and ≥80 cm for women according to the WHO Asia Pacific guidelines [14].

^i^ Systolic blood pressure ≥140 mmHg and/or diastolic blood pressure ≥90 mmHg and/or current use of anti-hypertensive medications according to the Joint National committee 7 criteria [15].
